# Como melhorar a mobilidade ativa em São Paulo, Brasil? Inquérito com lideranças de organizações não governamentais e com gestores públicos e privados

**DOI:** 10.1590/0102-311XPT117323

**Published:** 2024-06-14

**Authors:** Alex Antonio Florindo, Italo Vinicius Floriano de Paula, Douglas Roque Andrade, Flávia Mori Sarti, Jorge Mota, Maria Paula Santos, Margarethe Thaisi Garro Knebel, Rildo de Souza Wanderley, Leandro Martin Totaro Garcia

**Affiliations:** 1 Escola de Artes, Ciências e Humanidades, Universidade de São Paulo, São Paulo, Brasil.; 2 Faculdade de Saúde Pública, Universidade de São Paulo, São Paulo, Brasil.; 3 Faculdade de Medicina, Universidade de São Paulo, São Paulo, Brasil.; 4 Faculdade de Desporto, Universidade do Porto, Porto, Portugal.; 5 Centro de Investigação em Actividade Física, Saúde e Lazer, Universidade do Porto, Porto, Portugal.; 6 Laboratório para a Investigação Integrativa e Translacional em Saúde Populacional, Universidade do Porto, Porto, Portugal.; 7 Centre for Public Health, Queen’s University Belfast, Belfast, Northern Ireland.

**Keywords:** Caminhada, Ciclismo, Levantamento de Opinião, Organizações Não Governamentais, Walking, Bicycling, Surveys and Questionnaires, Nongovernmental Organizations, Caminata, Ciclismo, Encuestas y Cuestionarios, Organizaciones No Gubernamentales

## Abstract

Este estudo teve como objetivo descrever um inquérito quantitativo realizado com lideranças para investigar ações efetivas, viáveis e que podem ser testadas em modelos computacionais para informar políticas de promoção da mobilidade ativa, tendo como base a cidade de São Paulo, Brasil. Em 2022, foi realizado um inquérito *online* no contexto da pesquisa de Atividade Física e Ambiente do *Inquérito de Saúde de São Paulo*, acompanhada por representantes de organizações não governamentais, gestores públicos e de entidades privadas. Foi elaborado questionário com três perguntas com 13 alternativas de respostas sobre ações para promoção da caminhada ou uso de bicicleta. As lideranças deveriam selecionar até três alternativas a partir de seu potencial em termos de (1) efetividade; (2) viabilidade ou facilidade de implementação; e (3) desejo de realizar testes em modelos computacionais para informar políticas. O inquérito foi respondido por 18 lideranças de 16 instituições, sendo 13 (72%) mulheres e 12 (67%) representantes do terceiro setor, cuja média de idade era 48 anos, todos com nível superior de escolaridade. A redução da velocidade dos veículos motorizados foi a opção mais citada nas três questões. Outras ações citadas referem-se ao controle de circulação de veículos em regiões centrais, à segurança de pedestres, à diminuição das distâncias entre residências e locais de emprego, às campanhas educativas e à ampliação e melhoria de estruturas como ciclovias e calçadas. Os resultados são relevantes para apoiar a tomada de decisões baseadas em evidências na gestão pública e oferecer subsídios para a elaboração de modelos computacionais com vistas à promoção da mobilidade ativa.

## Abstract (en)

This study aimed to describe a quantitative survey conducted with leaders to investigate effective and feasible actions that can be evaluated in computational models to inform policies to promote active mobility based in the city of São Paulo, Brazil. In 2022, an online survey was conducted during the *Health Survey in São Paulo* (Physical Activity and Environment study), which is monitored by representatives of nongovernmental organizations and public and private sector managers. A questionnaire was elaborated with three questions with 13 alternative answers about actions to promote walking and/or cycling. Leaders should select up to three alternatives based on their potential regarding: (1) effectiveness; (2) feasibility or ease of implementation; and (3) desire to verify tests in computational models to inform policies. The survey was answered by 18 leaders from 16 institutions, comprising 13 (72%) women and 12 (67%) representatives of the third sector, whose average age was 48 years and all had complete higher education. Reducing the speed of motor vehicles was the most cited option in all three questions. Other actions mentioned refer to controlling the traffic of vehicles in central areas, improving pedestrian safety, reducing the distances between homes and places of employment, conducting educational campaigns, and expanding and enhancing structures such as bicycle lanes and sidewalks. The results are relevant to support evidence-based decision-making in public management and to provide subsidies for the development of computational models with a view to promoting active mobility.

## Resumen

Este estudio tuvo como objetivo presentar una encuesta cuantitativa realizada con líderes para investigar las acciones efectivas, viables y que puedan probarse en modelos informáticos para orientar las políticas que promuevan la movilidad activa en la ciudad de São Paulo, Brasil. En 2022 se realizó una encuesta en línea en el contexto de la *Encuesta de Salud de São Paulo* (Actividad Física y Medio Ambiente), que es monitoreada por representantes de organizaciones no gubernamentales, gestores públicos y entidades privadas. Se elaboró un cuestionario de tres preguntas con 13 respuestas alternativas sobre acciones para promover la caminata o el uso de la bicicleta. Los líderes podían seleccionar hasta tres alternativas en función de su potencial en términos de (1) efectividad; (2) viabilidad o facilidad de implementación; y (3) deseo de verificar las pruebas en modelos informáticos para orientar las políticas. La encuesta fue respondida por 18 líderes de 16 instituciones; de los cuales 13 (72%) eran mujeres y 12 (67%) representaban el tercer sector; la edad promedio de ellos fue de 48 años y todos contaban con educación superior. Reducir la velocidad de los vehículos de motor fue la opción más citada en las tres preguntas. Otras acciones mencionadas se refieren al control de la circulación de vehículos en las regiones centrales, la seguridad de los peatones, la reducción de las distancias entre los hogares y los lugares de trabajo, las campañas educativas y la expansión y mejora de estructuras como carriles bici y aceras. Los resultados son relevantes para apoyar la toma de decisiones basada en la evidencia en la gestión pública y ofrecer subsidios para la elaboración de modelos computacionales destinados a promover la movilidad activa.

## Introdução

A mobilidade ativa por meio do uso da bicicleta e da caminhada representa uma abordagem estratégica para promoção da saúde e prevenção de doenças no contexto das áreas urbanas nas cidades ^1^ ^,^ ^2^ ^,^ ^3^. Estudos epidemiológicos indicam que pessoas que caminham ou usam a bicicleta como forma de transporte têm risco diminuído de desenvolvimento de obesidade e doenças crônicas não transmissíveis. Ademais, a caminhada e o uso da bicicleta contribuem para diminuição da poluição do ar, diminuição da dependência do uso de veículos automotores, assim como contribuem para a melhoria da coesão social e reduzem a inatividade física e o comportamento sedentário ^2^ ^,^ ^4^ ^,^ ^5^ ^,^ ^6^ ^,^ ^7^.

No entanto, dados do Ministério da Saúde e estudos sobre tendência temporal da atividade física no Brasil ^8^ ^,^ ^9^ indicam redução progressiva da caminhada e do uso da bicicleta como forma de deslocamento ou transporte de 2006 a 2020 entre adultos residentes nas 26 capitais brasileiras e no Distrito Federal. O Município de São Paulo vem implementando importantes políticas públicas para a promoção da mobilidade ativa ao longo da última década, como o Plano Diretor em 2014, o Plano Municipal de Mobilidade Urbana em 2015 e o Estatuto do Pedestre em 2020 ^10^ ^,^ ^11^ ^,^ ^12^. Embora tais políticas tenham sido consideradas estratégias promissoras para promoção da saúde em estudo publicado no ano de 2022 ^13^, a definição do melhor conjunto de ações para promoção da mobilidade ativa ainda é um desafio para gestores em megacidades latino-americanas, como São Paulo.

Assim, uma estratégia importante para identificar as melhores ações em prol da promoção da mobilidade ativa em ambientes urbanos complexos consiste no trabalho colaborativo entre pesquisadores, gestores públicos e iniciativa privada, incluindo lideranças dos setores público, privado e terceiro setor. Ações governamentais bem planejadas apresentam potencial para maximizar os benefícios à sociedade e, simultaneamente, otimizar a utilização dos recursos públicos disponíveis. O levantamento das intervenções mais factíveis e efetivas para a mudança do cenário de incremento na prevalência de comportamentos sedentários e baixos níveis de atividades físicas da população deve ser baseado no conhecimento e na experiência de agentes envolvidos na prática das políticas públicas, como gestores públicos, formuladores de políticas e lideranças de organizações não governamentais (ONG) da cidade ^1^.

Ademais, a partir do conhecimento das lideranças, é possível propor a implementação de modelos computacionais que também contribuam como espaço de testes virtuais para a investigação dos impactos de diferentes iniciativas, bem como a comparação de possíveis externalidades positivas e negativas provenientes de uma variedade de ações de promoção da mobilidade ativa. O escopo dos modelos de simulação depende da definição dos cenários e das ações a serem testadas; assim, os modelos computacionais precisam refletir parte da realidade a ser reproduzida e testada, além de abordar intervenções possíveis de implementar em situações da vida real com oportunidade de validação dos resultados, sendo alinhados às necessidades populacionais e às condições objetivas vigentes aos tomadores de decisão em saúde pública ^14^.

Portanto, o objetivo deste artigo foi realizar um inquérito com lideranças de ONG, gestores públicos e privados vinculados à promoção da mobilidade ativa para investigar ações efetivas e factíveis para testes em modelos computacionais direcionados à priorização de estratégias de políticas públicas em favor do aumento da caminhada e do uso da bicicleta, tendo como base a cidade de São Paulo.

## Métodos

### Delineamento do estudo

Trata-se de estudo transversal conduzido no contexto do *Inquérito de Saúde de São Paulo* (ISA) - Atividade Física e Ambiente, que constitui uma coorte na cidade de São Paulo, cujo objetivo primário é verificar possíveis influências de mudanças ambientais no entorno das residências sobre as práticas de atividades físicas no lazer e como forma de deslocamento transporte, assim como investigar a influência do ambiente em outros desfechos secundários de saúde, como obesidade, doenças cardiovasculares e doenças mentais. Detalhes do protocolo da pesquisa foram publicados anteriormente ^15^.

A cidade de São Paulo tem, atualmente, uma população de 11.451.245 pessoas, de acordo com dados do último censo do Instituto Brasileiro de Geografia e Estatística (IBGE) ^16^. Em relação aos tipos modais de transporte utilizados pelos paulistanos, de acordo com dados da última pesquisa de *Origem e Destino* (OD) de 2017, 39,2% viajavam em transportes coletivos, 29,3% em transportes individuais, 30,7% faziam viagens a pé e 0,8% utilizavam bicicletas ^17^ ^,^ ^18^.

### Inquérito com lideranças

A pesquisa ISA - Atividade Física e Ambiente tem acompanhamento de lideranças da cidade de São Paulo, incluindo representantes de ONG, gestores públicos, gestores de entidades privadas e trabalhadores da saúde que têm relação com a temática do estudo. O objetivo principal da aproximação com diferentes lideranças é estabelecer e fortalecer vínculos interinstitucionais, intersetoriais e interprofissionais, com o intuito de potencializar a aplicação dos resultados para ações, programas e políticas direcionados à construção de ambientes favoráveis para promoção da atividade física, diminuição do comportamento sedentário e prevenção do excesso de peso e da obesidade. A aproximação com as lideranças foi iniciada no ano de 2019, com a elaboração de questionários, escolha dos primeiros representantes por conveniência e aplicação das primeiras entrevistas individuais para conhecer as expectativas e os interesses, envolvendo a identificação pessoal e institucional, objetivos da instituição, sugestões quanto à coleta de dados do estudo epidemiológico ISA - Atividade Física e Ambiente, estratégias para disseminação e utilização dos resultados e interesse em estabelecer vínculo com o estudo. Os resultados da primeira fase foram apresentados no Congresso Brasileiro de Atividade Física e Saúde em 2021 e publicados em forma de resumo nos anais do congresso na *Revista Brasileira de Atividade Física e Saúde* ^19^.

A etapa atual da pesquisa, apresentada neste artigo, iniciou-se em 2022 e teve como objetivo principal produzir evidências para informar a construção de um modelo baseado em agentes que permita testar ações estratégicas para promoção da mobilidade ativa na cidade de São Paulo, com vistas à futura implementação de políticas públicas. Assim, foi realizado um inquérito quantitativo junto às lideranças identificadas até o ano de 2022, para levantamento de conjunto de ações para promoção da caminhada e do uso de bicicletas em São Paulo. A pesquisa está sendo realizada em colaboração com pesquisadores do Centro de Saúde Pública da Universidade da Rainha em Belfast (Queen’s University Belfast), Irlanda do Norte, e do Centro de Investigação em Atividade Física, Saúde e Lazer da Universidade do Porto, Portugal, visando potencial adaptação do modelo a outras localidades.

O questionário utilizado no inquérito foi elaborado em cinco etapas: (1) discussão inicial com pesquisadores experientes na temática de mobilidade ativa e nas áreas de epidemiologia, economia, modelagem baseada em agentes e políticas públicas; (2) consulta aos documentos das políticas municipais de São Paulo, como o Plano Diretor de 2014, o Plano de Mobilidade Urbana e o Estatuto do Pedestre, para elaborar as questões e opções de respostas ^10^ ^,^ ^11^ ^,^ ^12^; (3) reunião com pesquisadores para discutir as questões e opções de respostas a serem incluídas no questionário; (4) consulta à segunda série de Desenho Urbano, Transporte e Saúde, publicada na revista *The Lancet Global Health* em 2022 ^3^, para a verificação da compatibilidade com as opções de respostas das questões; e (5) reunião final com pesquisadores para fechamento do questionário.

A versão final do questionário foi composta por três perguntas: (1) “Quais ações as lideranças acreditavam que seriam mais efetivas para aumentar os níveis de deslocamento ativo na cidade de São Paulo?”; (2) “Quais ações seriam mais viáveis/fáceis de serem implementadas?”; e (3) “Quais ações as lideranças gostariam de ver testadas em modelos de simulação computacional?”. Para cada pergunta, havia 13 opções de respostas idênticas e as lideranças poderiam escolher até três opções para cada questão (Quadro 1).

**Quadro 1:** Questionário final aplicado às lideranças no inquérito *online*. *Inquérito de Saúde de São Paulo* (ISA) - Atividade Física e Ambiente, 2022.

QUESTÕES APLICADAS
(1) Quais das ações abaixo você acredita que seriam mais efetivas para aumentar os níveis de deslocamento ativo na cidade de São Paulo? Entenda deslocamento ativo como a caminhada ou o uso da bicicleta
(2) Quais das ações abaixo você acredita que seriam mais viáveis/fáceis de implementar para mudar os níveis de deslocamento ativo na cidade de São Paulo? Entenda deslocamento ativo como a caminhada ou o uso da bicicleta
(3) Quais das ações abaixo você gostaria de ver testadas em um modelo de simulação para ter mais informações sobre o que fazer para mudar os níveis de deslocamento ativo na cidade de São Paulo? Entenda deslocamento ativo como a caminhada ou o uso da bicicleta
ALTERNATIVAS DE RESPOSTAS *
(A) Aumentar a acessibilidade a destinos comerciais a até 500 metros das residências
(B) Melhorar a distribuição de empregos pela cidade, deixando-os mais próximos às residências (por exemplo, a até 30 minutos de deslocamento a pé, de bicicleta ou transporte público)
(C) Aumentar a quantidade de faixas de pedestres e controladores de tráfego como lombadas e semáforos
(D) Aumentar a quantidade e melhorar a qualidade das calçadas de acordo com os padrões de pelo menos 1,5 metro de largura em toda a cidade
(E) Incentivar por meio de campanhas de mídia o uso da mobilidade a pé ou de bicicleta para deslocamentos cotidianos, como ir para o trabalho ou para a escola ou universidade
(F) Aumentar a quantidade e melhorar a qualidade das ciclovias, realizando ligações entre elas e com grandes estações de transportes públicos como trens, metrôs e terminais de ônibus
(G) Aumentar a quantidade de estacionamentos para bicicletas nas estações de trens, metrôs e terminais de ônibus
(H) Aumentar os programas de compartilhamento de bicicletas em toda a cidade com tarifas acessíveis
(I) Aumentar as possibilidades de entrada com bicicletas nas estações de transporte público, como trens e metrôs em horários de pico
(J) Aumentar a quantidade de estações de trens, metrôs e terminais de ônibus a até 1km de distância das residências
(K) Reduzir a velocidade dos veículos automotores
(L) Aumentar a restrição à circulação de veículos de transporte individual automotores nas regiões centrais da cidade, com taxações em horários de pico
(M) Melhorar a estrutura dos locais de trabalho para que as pessoas possam tomar banho caso se desloquem pedalando ou caminhando

* Cada liderança poderia escolher no máximo três alternativas de respostas para cada pergunta.

Fonte: elaboração própria.

Em seguida, o questionário foi reproduzido em um formulário eletrônico no aplicativo Google Forms (https://www.google.com/forms/) para coletar dados por meio de autorresposta *online*.

Posteriormente, foram identificadas por conveniência pelos pesquisadores responsáveis 46 lideranças de instituições formadas por pessoas que representavam ONG, gestores públicos do campo da saúde, transporte, lazer e esporte, e gestores de entidades privadas, como empresas de aplicativos de automóveis e de aluguel de bicicletas. O convite foi feito inicialmente por carta padronizada encaminhada por e-mail entre 8 de novembro e 15 de dezembro de 2022.

As lideranças poderiam ser de outras localidades, isto é, não precisariam ser ligadas diretamente ao Município de São Paulo, e os pesquisadores poderiam convidar mais de uma liderança da mesma instituição caso julgassem conveniente.

Após o primeiro convite, foram enviados novos e-mails como lembrete a quem não havia respondido ao questionário. Também foram realizadas tentativas de contato telefônico ou via aplicativo WhatsApp. O formulário ficou disponível para preenchimento até o final do mês de dezembro de 2022.

As análises deste artigo foram realizadas por meio da descrição das características individuais dos respondentes, como sexo, idade, nível de escolaridade, função dentro da instituição, tipo de instituição e setor que representavam. Em seguida, foram analisadas frequências das respostas para cada uma das três questões: (1) ações mais efetivas; (2) ações mais fáceis ou viáveis; e (3) ações que gostariam de ver testadas em modelos de simulação. Foram calculadas as frequências relativas e posteriormente estabelecidas as posições das três respostas mais frequentes em cada questão.

### Questões éticas

O estudo foi aprovado pelos comitês de ética em pesquisa da Escola de Artes, Ciências e Humanidades da Universidade de São Paulo (protocolo nº 10396919.0.0000.5390) e da Secretaria Municipal de Saúde de São Paulo (protocolo nº 10396919.0.3001.0086).

## Resultados

Foram obtidas respostas de 18 lideranças (39% do total do cadastro), sendo 13 lideranças do sexo feminino (72%), com média de idade 48 anos (desvio padrão [DP] = 11 anos) e todas com nível superior de escolaridade. As lideranças possuíam cargos ou funções como cofundadores e diretores, gerentes e assessores.

As lideranças que responderam representavam 16 instituições, sendo que duas (11%) lideranças representavam o segundo setor social (instituições privadas com fins lucrativos), sendo uma voltada ao planejamento urbano e outra à mobilidade por *bikesharing*. Doze (67%) lideranças representavam o terceiro setor social (instituições sem fins lucrativos), sendo que cinco atuavam na área de planejamento urbano e mobilidade ativa, quatro atuavam especificamente no cicloativismo e três atuavam especificamente incentivando a caminhada. Quatro (22%) lideranças representavam o setor público, sendo duas na área da saúde, uma na área de transportes e uma na área de esportes e lazer.

A síntese das respostas sobre ações de maior efetividade para aumentar a mobilidade ativa na cidade de São Paulo segundo as lideranças indicou priorização da redução da velocidade dos veículos motorizados (19%), seguida pela melhoria da distribuição de empregos pela cidade, isto é, promover maior proximidade entre locais das residências e de trabalhos (p.ex.: até 30 minutos de deslocamento a pé, de bicicleta ou de transporte público) (17%). Em terceiro, houve empate entre o aumento da quantidade e a melhoria da qualidade das calçadas, de acordo com os padrões de pelo menos 1,5m de largura (13%), e o aumento da quantidade e a melhoria da qualidade das ciclovias, incluindo ligações entre elas e com grandes estações de transporte público, como trem, metrô e terminais de ônibus (13%) (Tabela 1).

**Tabela 1:** Opinião das lideranças sobre ações de maior efetividade, viabilidade ou facilidade de implementação e adequação para testes em modelos computacionais para promoção da mobilidade ativa na cidade de São Paulo, Brasil, 2022.

Ações para a promoção da mobilidade ativa (caminhada ou uso de bicicleta)	Ações mais efetivas		Ações mais fáceis ou viáveis		Ações que gostaria de ver testadas	
	%	Posição	%	Posição	%	Posição
(A) Aumentar a acessibilidade a destinos comerciais a até 500 metros das residências	9	4º	2	8º	4	5º
(B) Melhorar a distribuição de empregos pela cidade, deixando-os mais próximos às residências (por exemplo, a até 30 minutos de deslocamento a pé, de bicicleta ou transporte público)	17	2º	2	8º	9	3º
(C) Aumentar a quantidade de faixas de pedestres e controladores de tráfego como lombadas e semáforos	2	7º	11	3º	7	4º
(D) Aumentar a quantidade e melhorar a qualidade das calçadas de acordo com os padrões de pelo menos 1,5 metro de largura em toda a cidade	13	3º	9	4º	7	4º
(E) Incentivar por meio de campanhas de mídia o uso da mobilidade a pé ou de bicicleta para deslocamentos cotidianos, como ir para o trabalho ou para a escola ou universidade	6	5º	17	2º	2	6º
(F) Aumentar a quantidade e melhorar a qualidade das ciclovias, realizando ligações entre elas e com grandes estações de transportes públicos como trens, metrôs e terminais de ônibus	13	3º	11	3º	15	1º
(G) Aumentar a quantidade de estacionamentos para bicicletas nas estações de trens, metrôs e terminais de ônibus	9	4º	9	4º	11	2º
(H) Aumentar os programas de compartilhamento de bicicletas em toda a cidade com tarifas acessíveis	2	7º	2	8º	9	3º
(I) Aumentar as possibilidades de entrada com bicicletas nas estações de transporte público, como trens e metrôs em horários de pico	2	7º	6	7º	4	5º
(J) Aumentar a quantidade de estações de trens, metrôs e terminais de ônibus a até 1km de distância das residências	6	5º	2	8º	2	6º
(K) Reduzir a velocidade dos veículos automotores	19	1º	20	1º	15	1º
(L) Aumentar a restrição à circulação de veículos de transporte individual automotores nas regiões centrais da cidade, com taxações em horários de pico	4	6º	7	6º	15	1º
(M) Melhorar a estrutura dos locais de trabalho para que as pessoas possam tomar banho caso se desloquem pedalando ou caminhando	0	8º	2	8º	0	7º

Fonte: elaboração própria.

Quanto às ações de maior viabilidade ou facilidade de implementação para mudanças nos deslocamentos ativos (Tabela 1), as opções mais citadas foram: a redução da velocidade dos veículos motorizados (20%); o incentivo por meio de campanhas de mídia em favor da caminhada ou do uso de bicicleta para deslocamentos cotidianos, como ir para o trabalho, escola ou para a universidade (17%); e, empatadas em terceiro lugar, o aumento da quantidade de faixas de pedestres e controladores de tráfego como lombadas e semáforos (11%) e o aumento da quantidade e a melhoria da qualidade das ciclovias, realizando ligações entre si e com grandes estações de transportes públicos, como de trens, metrôs e terminais de ônibus (11%).

Em termos das ações que as lideranças relataram que gostariam de ver testadas em modelos computacionais (Tabela 1), houve um empate das seguintes ações em primeiro lugar: redução da velocidade dos veículos motorizados (15%), aumento da restrição à circulação de veículos de transporte individual motorizados nas regiões centrais da cidade, com taxações em horários de pico (15%) e aumento da quantidade e melhoria da qualidade das ciclovias, realizando ligações entre elas e com grandes estações de transportes públicos, como trens, metrôs e terminais de ônibus (15%).

## Discussão

As lideranças entrevistadas neste estudo apontaram diferentes ações que podem ser efetivas, viáveis e que gostariam de ver testadas em modelos computacionais para promover a caminhada ou o uso da bicicleta na cidade de São Paulo. Considerando inicialmente as ações ligadas diretamente à segurança no trânsito, que podem servir tanto para a promoção da caminhada como para o uso de bicicleta, a redução da velocidade de veículos motorizados foi selecionada como a primeira ação de maior efetividade, viabilidade e facilidade de implementação e que as lideranças gostariam de ver testadas em modelos computacionais.

É interessante ressaltar que a redução da velocidade de veículos automotores já está incluída no Plano Global pela Segurança no Trânsito, proposto pela Organização Mundial da Saúde (OMS) e pela Organização das Nações Unidas (ONU) ^20^. É importante ressaltar que o Plano de Metas da Prefeitura do Município de São Paulo, proposto até 2024, prevê reduzir o índice de mortes no trânsito para 4,5 por 100 mil habitantes, com ações para a redução de velocidade de veículos automotores de 50km/h para 40km/h em 24 vias e implantação de minirrotatórias ^21^.

O relatório anual de acidentes de trânsito, publicado pela Companhia de Engenharia de Tráfego da cidade de São Paulo, aponta que houve redução de 47% no número de acidentes fatais entre pedestres de 2012 a 2021 ^22^. Ressalta-se que os pedestres são os mais atingidos por fatalidades no trânsito de São Paulo, representando 35% dos acidentes fatais em 2021, seguidos dos motociclistas ^22^. Acredita-se que as ações de controle de velocidade contribuíram para a redução dos acidentes, mesmo com aumento na frota de veículos motorizados em São Paulo, que foi de 3.781.040, em 2006, para 6.094.036, em 2022 ^23^. A redução da velocidade dos veículos automotores tem sido implementada em algumas áreas da cidade em cumprimento às disposições do *Decreto nº 58.717*, de 17 de abril de 2019, que aborda o Plano de Segurança Viária de São Paulo ^24^. Esse decreto embasou o Programa Áreas Calmas, que tem como objetivo primário aprimorar a segurança no trânsito, incluindo ações de implementação de dispositivos para moderação do tráfego, como travessias elevadas, avanços de calçadas, lombadas, rotatórias, além da redução do limite máximo de velocidade para 30km/h ^25^.

Outra ação importante relacionada ao maior controle de veículos automotores e à promoção tanto da caminhada como com o uso da bicicleta, que esteve entre as primeiras que as lideranças gostariam de ver testadas em modelos computacionais, foi o aumento da restrição à circulação de veículos de transporte individual em regiões centrais, com taxações em horários de pico. Essa ação pode ser uma alternativa para diminuir os congestionamentos de veículos automotores e, ao mesmo tempo, incentivar o uso de transportes públicos coletivos, que também se associam à caminhada e ao uso de bicicleta. No entanto, é importante que se tenha aumentos de forma equânime de grandes estações de transportes públicos por toda a cidade de São Paulo ^26^. Estudos epidemiológicos realizados com adultos das cidades de São Paulo e Curitiba (Paraná) têm mostrado que pessoas que residem a até 1km de grandes estações de transportes públicos e coletivos têm mais chances de caminhar ou utilizar a bicicleta para deslocamentos ^27^ ^,^ ^28^.

A cidade de Londres (Inglaterra) foi pioneira no mundo na adoção de pedágios urbanos para veículos motorizados individuais desde 2003 ^29^. No caso do Brasil, o plano elaborado a partir da Política Nacional de Mobilidade Urbana permite que municípios brasileiros adotem áreas e horários de circulação restrita ou controlada. No caso de São Paulo, um estudo identificou custos crescentes dos congestionamentos de veículos automotores entre 2002 e 2012, com substanciais prejuízos aos habitantes do município ^30^. A implantação de pedágios urbanos é uma das ações indicadas para minimizar o problema, juntamente com várias outras mudanças que deveriam ocorrer, como investimentos em ampliação de transportes por meio de trens e metrô ^30^.

Quanto às estruturas de segurança dos pedestres e às relações com a promoção da caminhada como forma de transporte, o aumento do número de calçadas, priorizando estruturas de qualidade de acordo com normas estabelecidas, a ampliação do número de ciclovias, melhorando as ligações delas com grandes estações de transporte públicos, e a expansão da quantidade de faixas de pedestres e controladores de tráfego, como lombadas e semáforos, foram ações que ficaram em terceiro entre as mais citadas como mais viáveis, fáceis e efetivas para promover a mobilidade ativa.

Assim como a diminuição da velocidade dos veículos automotores, essas ações são estratégias diretamente relacionadas à segurança de pedestres ^20^. O Estatuto do Pedestre e o Plano de Segurança Viária preconizam várias ações para o aumento e a melhoria das faixas de pedestres e dos controles semafóricos ^12^. Entretanto, uma análise realizada pelo associação Cidadeapé, em 2020, indicou que a falta de faixas de pedestres e de controles semafóricos ainda não está adequada para a segurança, impedindo a caminhada em várias regiões da cidade ^31^. Além disso, muitos controladores de tráfego não funcionam corretamente e eles ficam pouco tempo abertos para os pedestres. Estudo realizado com idosos paulistanos mostrou que o tempo dos semáforos para os pedestres, em cruzamentos de ruas e avenidas, é insuficiente, levando-se em conta a velocidade de caminhada média da população idosa ^32^. Ações como o Programa de Proteção ao Pedestre vêm sendo propostas pela Prefeitura do Município de São Paulo, com o objetivo de resgatar o respeito e aumentar a segurança dos caminhantes ^33^.

Quanto às calçadas, que contribuem diretamente para a promoção da caminhada, apesar de a Prefeitura do Município de São Paulo conter, no seu plano de metas, a ampliação desse atributo ^21^, um relatório publicado pelo Centro de Estudos da Metrópole, em 2019, identificou substancial desigualdade na condição dessas estruturas em São Paulo ^34^. Algumas subprefeituras de regiões periféricas, com menor nível socioeconômico, apresentaram menores medianas em termos de largura de calçadas, em comparação com regiões centrais de maior nível socioeconômico, como a região oeste da cidade ^34^. Além disso, identificou-se maior largura nas calçadas em regiões com maior proporção de população de cor de pele branca ^34^. Portanto, a promoção da caminhada como forma de mobilidade ativa deve ser baseada no cumprimento da normatização preconizada no Estatuto do Pedestre ^12^, ou seja, de forma equânime no Município de São Paulo e seguindo as ações prioritárias do Observatório de Mobilidade Segura criado pela Prefeitura do Município de São Paulo em 2020 ^35^.

Quanto às ações ligadas a estruturas para o uso de bicicletas como meio de transporte, aumentar a quantidade e melhorar a qualidade de ciclovias, realizando ligações entre elas e com grandes estações de transportes públicos, esteve em primeiro lugar como a ação mais citada que as lideranças gostariam de ver testadas em modelos computacionais e em terceiro entre as ações mais fáceis e viáveis e mais efetivas para a mobilidade ativa. As ciclovias são estruturas que podem favorecer diretamente o transporte por meio de bicicletas e os governos do Município de São Paulo vêm adotando políticas de aumento da extensão de ciclovias a partir do Novo Plano Diretor em 2014. Estudo mostrou que, entre 2015 e 2020, houve um aumento de 67,7% na extensão de ciclovias no município, passando de 436,3km para 731,4km ^26^. Entretanto, identificou-se que a maior parte das ciclovias ainda se concentram em áreas de maior nível socioeconômico ^26^. Estudo epidemiológico realizado com população adulta, na cidade de São Paulo, indicou maior probabilidade de uso da bicicleta como forma de deslocamento ou transporte entre pessoas que residem em áreas com ciclovias a até 500m de suas moradias ^36^. Além disso, pessoas com residências a até 1.500m de distância de estações de trem ou metrô também tiveram maior probabilidade de usar a bicicleta como transporte ^36^. No entanto, quando se verificou a relação entre as ciclovias e as estações de trem e metrô de forma conjunta nos *buffers* de 1.500m ao redor de residências, não foram obtidos resultados significativos para o aumento das chances de se usar a bicicleta como deslocamento ou transporte, apontando a necessidade de melhoria nas ligações entre ciclovias em relação às estações de transporte público.

O incentivo por meio de campanhas de mídia para a conscientização da população sobre a importância da mobilidade a pé ou de bicicleta para deslocamentos cotidianos, como ir para os locais de trabalhos ou estudos, esteve na segunda posição entre as ações mais fáceis e viáveis para promover a mobilidade ativa. Em Brasília (Distrito Federal), uma ação para aumentar o respeito às faixas de pedestres reduziu em 81,5% o número de atropelamentos em 25 anos ^37^. A campanha tem como ponto central a educação no trânsito e envolve ações em escolas e comunicação em jornais, com participação da população, da mídia e dos governos ^38^. Rodrigues ^39^ defende que campanhas de educação no trânsito devem ser um processo de aprendizagem contínuo para diferentes faixas etárias, inserido nos diversos níveis de educação formal, desde a infância até a fase adulta, ou seja, campanhas não podem ser limitadas somente à transmissão de informações para uma determinada faixa etária em período limitado de tempo. Além disso, o envolvimento das famílias na educação no trânsito também tem papel relevante na redução de eventos adversos no transporte e no incentivo à mobilidade ativa como promoção cultural, como o trabalho que é realizado pela organização Carona a Pé ^40^.

A melhoria da distribuição de empregos pela cidade, deixando-os mais próximos às residências foi a segunda ação que as lideranças consideraram mais efetiva para a promoção da mobilidade ativa em São Paulo. Essa é uma mudança estrutural complexa, mas que têm relação direta com cidades saudáveis e sustentáveis, na medida em que colaboram para diminuir as distâncias dos deslocamentos das residências aos locais de trabalho e de estudos, de forma que as pessoas tenham mais oportunidades de utilizar a caminhada e a bicicleta, além de terem mais tempo livre ^41^ ^,^ ^42^. De acordo com Giles-Corti et al. ^3^, em editorial publicado na segunda série de desenho urbano, transporte e saúde da revista *The Lancet Global Health*, indicadores como porcentagem da população com emprego a menos de 30 minutos de suas residências e proporção de empregos por residências em uma determinada área da cidade são importantes para promover a caminhada e o uso da bicicleta como forma de deslocamento ou transporte.

Além disso, é importante ressaltar que regiões com maior mistura de destinos, incluindo proximidade de padarias, supermercados, estações de trem ou metrô, principalmente a até 1km das residências, proporcionam mais chances de caminhada como forma de deslocamento ou transporte ^27^. No entanto, os paulistanos ainda estão muito distantes de dados ideais de aproximação das suas residências até os seus locais de trabalho ou estudo. Dados de inquérito coordenado pela Rede Nossa São Paulo ^43^ com amostra representativa de indivíduos adultos, realizado no ano de 2021, indicaram que somente 17% das pessoas gastavam até 30 minutos nos seus deslocamentos diários, apontando substanciais desigualdades entre as regiões do Município de São Paulo. Alguns países já estão adotando o conceito de “cidades de 30 minutos” ^44^, que significa que os deslocamentos para a maioria das tarefas do cotidiano das pessoas (como trabalho, estudo e compras) deveriam ter, no máximo, 30 minutos. Outros pesquisadores e gestores propõem o conceito de “bairros de 15 minutos”, nos quais a maioria dos deslocamentos poderiam ocorrer por meio de caminhada ou uso de bicicleta, principalmente após a pandemia de COVID-19 ^45^ ^,^ ^46^ ^,^ ^47^. Portanto, a ação de aproximação das residências aos locais de trabalho e de estudo é extremamente complexa, pois exige planejamento intersetorial, envolvendo os setores de desenvolvimento urbano, transporte e saúde ^13^.

**Figura 1:**
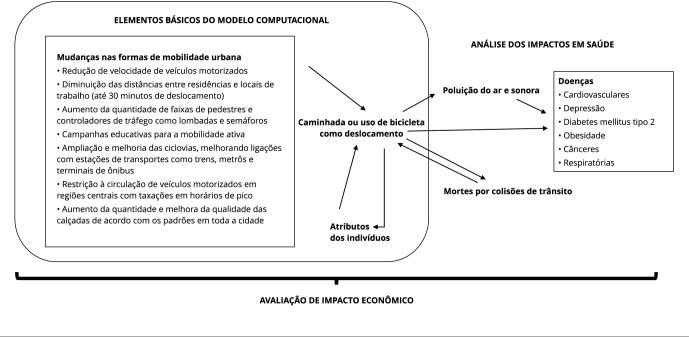
Proposta de modelo de análises computacionais baseado nas respostas das lideranças.

As estratégias de promoção da caminhada e do uso da bicicleta como mobilidade ativa elencadas neste estudo são passíveis de implementação por diversas alternativas, especialmente em uma metrópole complexa como São Paulo. A identificação de conjuntos de ações de maior efetividade e menor custo, assim como potenciais externalidades positivas e negativas de cada alternativa, é uma etapa importante na tomada de decisões baseadas em evidências de gestão pública. Modelos computacionais que permitam realizar simulações para diferentes ações, implementadas de forma isolada ou conjunta, constituem uma importante ferramenta de apoio para a formação da agenda, a formulação e a implementação de políticas públicas com maior chance de sucesso em saúde pública ^14^. A partir dos resultados deste estudo, seria possível implementar modelos computacionais, como modelos baseados em agentes, conforme preconizado na Figura 1.

Fonte: elaboração própria.

Considerando-se possíveis as mudanças nas formas de mobilidade urbana, como sugerido pelas lideranças, é possível verificar os efeitos na mobilidade ativa (como caminhada e uso de bicicleta), que são atributos dos agentes no modelo (pessoas). Posteriormente, será possível calcular efeitos das diferentes ações de mobilidade ativa sobre as dimensões ambientais (como poluição do ar e sonora) e mortalidade no trânsito. Finalmente, será possível verificar possíveis efeitos em doenças e os impactos econômicos (como custos de tratamento de violência no trânsito e morbidades em comparação com custos das intervenções).

Testes computacionais podem apoiar a seleção dos melhores locais e condições ideais para otimizar os benefícios sociais, ambientais, econômicos e de saúde de uma determinada ação, assim como minimizar os riscos e identificar as melhores combinações de estratégias para gerar sinergias. A possibilidade de testar intervenções em um ambiente virtual, como locais ou formas de implementação de maior custo-efetividade para delimitar áreas de trânsito calmo (com velocidades de até 30km/h) ou mudanças na distribuição dos empregos da cidade, representa uma metodologia rápida e com menor custo para gerar evidências quando testes no mundo real são inviáveis em termos éticos ou econômicos, pois envolvem mudanças na vida das pessoas e, em geral, necessitam de anos para a execução e observação de seus resultados. Ademais, podem contribuir com o Plano de Metas da Prefeitura do Município de São Paulo e, utilizando-se de respostas obtidas por meio da opinião de lideranças, é possível delimitar parâmetros realistas aos modelos de simulação computacional.

Este estudo apresenta algumas limitações, particularmente em relação ao tamanho amostral. Pesquisas subsequentes devem ampliar a amostra para a representação de lideranças em São Paulo e em outras cidades brasileiras, a fim de verificar potenciais diferenças na opinião e nas necessidades populacionais para paulistanos e em outras regiões do país. Além disso, houve predominância de respostas de lideranças do terceiro setor (67%), superestimando a opinião dessa representação e subestimando opiniões de gestores públicos e privados, o que poderia mudar as escolhas das ações prioritárias. Ressalta-se, também, que foi uma amostra escolhida por conveniência, de acordo com os registros das lideranças que, de alguma forma, tiveram contato com a pesquisa ISA - Atividade Física e Ambiente. Novos estudos poderiam considerar amostras maiores e aleatórias que pudessem representar diferentes lideranças de diferentes setores. Outra limitação do estudo refere-se à ausência de teste de medidas de reprodutibilidade do questionário antes da aplicação. Entretanto, é importante destacar que o questionário foi amplamente discutido em duas rodadas de debate entre pesquisadores com experiência em epidemiologia e políticas públicas relacionadas ao tema, assim como foi baseado em um conjunto robusto de evidências sintetizadas em documentos oficiais de instituições locais e internacionais de relevância no campo da mobilidade ativa, resultando em maior validade do conteúdo do instrumento.

## Conclusão

Este estudo realizou levantamento de pelo menos três ações que lideranças de organizações não governamentais, gestores públicos e de entidades privadas consideraram efetivas, fáceis e viáveis ou que gostariam de ver testadas em modelos computacionais para a promoção da caminhada e do uso da bicicleta em São Paulo. Identificou-se que ações direcionadas ao controle da velocidade de veículos automotores, controle de circulação de veículos em algumas regiões com taxações, ações para a segurança de pedestres e para a diminuição das distâncias entre residências e locais de trabalho ou de estudos, campanhas educativas e para a ampliação e melhoria de estruturas como ciclovias e calçadas são consideradas estratégias importantes para a mobilidade ativa. Este estudo apresenta resultados inovadores, principalmente porque foram obtidos com base na opinião de lideranças que trabalham com saúde, lazer e esporte, ambiente e transporte.

Ressalta-se que alguns desses resultados condizem com o Plano de Metas da Prefeitura do Município de São Paulo estabelecido até o ano de 2024 ^21^. Nesse sentido, modelos computacionais de simulação podem apoiar a tomada de decisões baseadas em evidências na gestão pública, tendo em vista a complexidade da questão no contexto urbano de São Paulo. Acredita-se que a caminhada e o uso de bicicleta devem ser colocados como prioridades nas formas de mobilidade, dada a relação que têm com cidades saudáveis e sustentáveis, com as metas de desenvolvimento sustentável preconizadas pela ONU ^48^ e com vistas à criação de sociedades ativas, ambientes e sistemas ativos, como preconizado pelo Plano de Ação Global de Atividade Física da OMS ^49^.
